# Haemanthidine-Containing Alkaloid Fraction from *Crinum scabrum* as a Natural Therapeutics for Chagas Disease

**DOI:** 10.1021/acsomega.5c10218

**Published:** 2025-12-25

**Authors:** Jennifer Blandón Pardo, Lorraine Martins Rocha Orlando, Leonardo da Silva Lara, Mirian Claudia de Souza Pereira, Natália Ferreira de Sousa, Luciana Scotti, Marcus Tullius Scotti, Warley de Souza Borges

**Affiliations:** † Natural Products Research Group, Department of Chemistry, Federal University of Espírito Santo, UFES, Vitória 29075-910, ES, Brazil; ‡ Cellular Ultrastructure Laboratory, Oswaldo Cruz Institute, Fiocruz, Rio de Janeiro 21040-900, Brazil; § Postgraduate Program in Bioactive Natural and Synthetic Products, Federal University of Paraíba, Campus I, Cidade Universitária, João Pessoa 58051-900, PB, Brazil

## Abstract

*Crinum scabrum*, an underexplored species and potential source of the bioactive alkaloid haemanthidine, was investigated. A crude methanolic extract was prepared and subjected to acid–base extraction to obtain an alkaloid-enriched fraction. Four alkaloids were isolated and purified; structural characterization via ^1^H and ^13^C NMR spectroscopy, coupled with literature comparison, identified them as haemanthidine/6-*epi*-haemanthidine (**C1**/**2**), crinamine (**C3**), and hamayne (**C4**). The extracts and purified compounds were screened against *Trypanosoma cruzi* (amastigote and trypomastigote forms) and Vero host cells. Fraction III exhibited potent activity against intracellular amastigotes (IC_50_ = 0.88 ± 0.08 μg mL^–1^), comparable to benznidazole (IC_50_ = 0.75 ± 0.07 μg mL^–1^), with exceptional selectivity (SI > 568.2). Critically, no extract and fractions inhibited trypomastigotes (IC_50_ > 500 μg mL^–1^), mirroring benznidazole’s stage-specific limitation. For intracellular amastigotes, purified **C1/C2** (IC_50_ = 6.05 ± 0.53 μM) and **C3** (IC_50_ = 5.79 ± 0.17 μM) showed the highest potency. All compounds exhibited low cytotoxicity (CC_50_ > 200), yielding exceptional selectivity indices (SI > 80). Additionally, through integrated computational target prediction (PharmMapper) and molecular docking simulations, the study identified and validated the inhibition of *T. cruzi* GAPDH and Cruzain as key mechanisms of action for the bioactive alkaloids. These findings position *C. scabrum* alkaloid-enriched fraction as a promising low-toxicity alternative for chronic Chagas disease, warranting *in vivo* validation and metabolomic profiling to elucidate bioactive cofactors.

## Graphical abstract

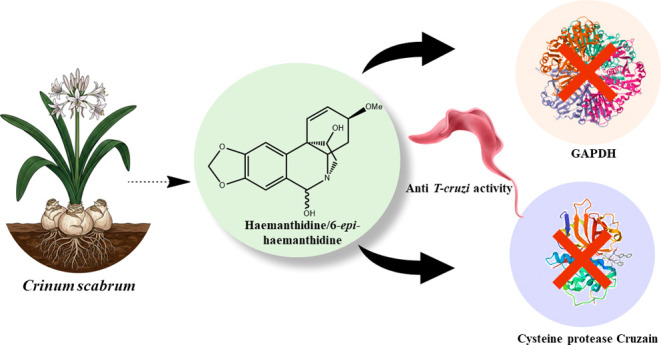

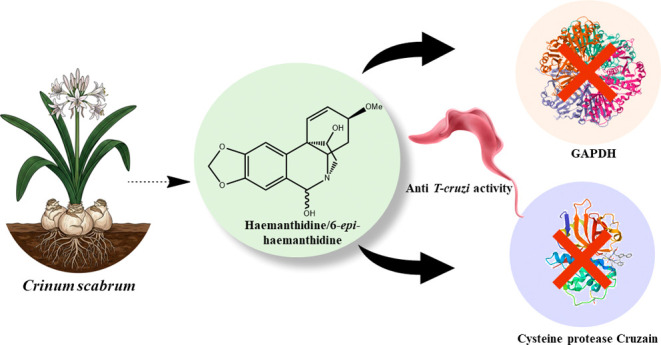

## Introduction

Chagas disease (ChD), also known as American trypanosomiasis, remains a chronic and life-threatening parasitic illness caused by the protozoan *Trypanosoma cruzi*.^1^ As a neglected tropical disease (NTD), it imposes a disproportionate public health burden across Latin America, primarily transmitted through triatomine insect vectors.^2–4^ Currently, the World Health Organization (WHO) estimates indicate over 7 million global infections, with Brazil, a hyperendemic epicenter, harboring approximately 1.2 million cases and 6000 annual deaths.^5,6^

Despite progress in vector control, outdated seroprevalence data (4.22% in rural areas)^7^ and systemic underreporting obscure the true epidemiological toll.^8^

Compounding this crisis, nonvector transmission routes through congenital routes,^9^ blood transfusions,^10^ organ transplants,^11^ and contaminated food^12^ have globalized ChD, while therapeutic limitations persist. Benznidazole (Bz) and nifurtimox exhibit severe toxicity, variable efficacy in chronic stages, and limited accessibility.^13^

This therapeutic void has intensified the search for novel anti*-*trypanosomal agents, with natural products offering structurally diverse scaffolds for the development of new drugs. Notably, Amaryllidaceae alkaloids (AAs) have emerged as promising candidates due to their multifaceted action against *T. cruzi*. These compounds compromise parasite viability through the plasma membrane disruption, inducing osmotic lysis and cellular dysfunction by damaging membrane integrity.^14^ Concurrently, they alter mitochondrial membrane potential, uncoupling oxidative phosphorylation and depleting ATP reserves essential for parasite survival, a vulnerability particularly pronounced in energy-dependent amastigotes.^14^ Further, AAs inhibit key parasitic enzymes, such as acetylcholinesterase and trypanothione reductase, disrupting critical metabolic pathways.^15^ While these mechanisms are well-supported, it is important to note that specific studies confirming the lethal generation of oxidative stress by AAs, through the production of Reactive Oxygen Species (ROS) that overwhelm *T. cruzi*’s antioxidant defenses and cause irreversible macromolecular damage, are currently lacking. However, given that mitochondrial dysfunction is a known consequence of some AA treatments, a secondary increase in ROS production remains a plausible,^16^ albeit unconfirmed, contributing factor to their antiparasitic effects. Importantly, certain AAs exhibit stage-specific activity, as observed with hippeastrine,^17^ which preferentially targets intracellular amastigotes by interfering with differentiation and proliferation processes. This mechanistic diversity positions AAs as compelling scaffolds for overcoming drug resistance in ChD.^18^

Building on the pharmacological foundation AAs with documented efficacy antiparasitic against *T. cruzi*, our research group has conducted phytochemical studies on *Crinum scabrum* (Amaryllidaceae), a poorly explored species, identifying it as a potent source of haemanthidine.^19^ This crinane-type alkaloid’s key interest lies in its ability to disrupt mitochondrial function, a mechanism that aligns precisely with the AA-mediated action described against *T. cruzi*.^20^

This study sought to validate the potential of *C. scabrum* as a strategic candidate for the development of an anti-Chagas drug. This was achieved through the *in vitro* evaluation of the anti-*T. cruzi* potential of the methanolic extract, and its polar fractions, and the isolated compounds, both individually and in combination with benznidazole. Finally, *in silico* studies were conducted to determine and confirm the possible mechanism of action of these compounds.

## Methodology

### Plant Material

The leaves and bulbs of *C. scabrum* were collected at Goiandira, Goiás, Brazil (18°03′34.5″ S, 48°07′49.8″ W) in October 2016 (SisGen register: A012E5E). The plant was identified by Antônio Campos-Rocha and a Voucher specimen (VIES 24824) was deposited at the Herbarium from Federal University of Espírito Santo, Vitória, Espírito Santo, Brazil (VIES – UFES).

#### Alkaloid Extraction, Isolation, and Identification

The crude methanolic extract (E.MeOH; 163.21 g) from the leaves and bulbs of *C. scabrum* and its subsequent acid–base extraction was performed as previously described,^19^ to yield four fractions (I, II, III, and IV). The fraction III was concentrated (8.77 g) and fractionated by silica-gel column chromatography by eluting with a gradient of hexane/(CH_3_)_2_CO (from 100:0 to 0:100, v/v) to obtain 21 fractions (III_1–21_). The III_17_ yielded compound **1/2** (2.9645 g, 0.086% w/w relative to dry plant material); III_20_ compound **3** (1.402 g, 0.041 w/w), and III_2**1**_ compound **4** (0.210 g, 0.06% w/w). The compounds’ structures were identified by nuclear magnetic resonance (^1^H NMR) and data comparison with the literature.

### Biological Assays

#### Cell Culture

Vero cells, epithelial cells derived from the kidney of the African green monkey, were cultured in RPMI 1640 medium supplemented with 10% fetal bovine serum (FBS). The cell monolayers were dissociated using a trypsin and EDTA solution (0.025%) and placed in 150 cm^2^ flasks or white 96-well plates. The cells were used to cultivate trypomastigote forms of *T. cruzi* and in cytotoxicity and phenotypic screening assays.

#### Parasites

The phenotypic screening assay used *T. cruzi* clone Dm28c, which was genetically modified to express luciferase (Dm28c-Luc) by Dr. Cristina Henriques.^21^ Trypomastigotes were obtained from the supernatant of infected Vero cell cultures on the fourth day postinfection (4 dpi) and used to analyze antiparasitic activity.

#### Cytotoxicity Assay

Vero cells, seeded at a density of 1.5 × 10^4^ cells/well in a white 96-well plate, were cultivated for 24 h at 37 °C in RPMI 1640 medium with 10% FBS. The cell monolayers were treated with E.MeOH of *C. scabrum* and its fractions (I, III, IV) at concentrations ranging from 62.5 to 500 μg/mL for 72 h at 37 °C. Additionally, isolated alkaloids (**C1/2**, **C3**, and **C4**) were administered to the Vero cells for 72 h at concentrations from 15.62 to 500 μM. Cell viability post-treatment was evaluated by measuring ATP levels using CellTiter-Glo reagent (Promega Corporation, Madison, WI), with luminescence recorded on a Glomax microplate reader (Promega Corporation, Madison, WI). Controls were performed with dimethyl sulfoxide (DMSO) at concentrations below 1% and benznidazole (Bz) at varying concentrations (ranging from 15.62 to 500 μM, as the reference drug. The half-maximal cytotoxicity concentration (CC_50_) was calculated using Prism GraphPad software (version 8.2.1). The experiments were conducted in triplicate, with each assay performed in duplicate.

#### Antiparasitic Activity

The trypanocidal activity of the E.MeOH from *C. scabrum*, along with its fractions (I, III, IV), isolated alkaloids (**C1/2**, **C3** and **C4**) and Bz were investigated against both stages of *T. cruzi* (trypomastigote and intracellular amastigote). For the viability assays of trypomastigotes, a 24 h exposure to a range of concentrations (0.22 to 500 μg mL^–1^ for the crude extract and fractions; 0.041 to 100 μM for the isolated alkaloids and Bz) was performed at 37 °C. Following treatment, luminescent-based viability was determined by adding luciferin (300 μg mL^–1^), the substrate for luciferase, and measuring the resulting luminescence with a Glomax microplate reader. The 50% inhibitory concentration (IC_50_) was calculated to determine the concentration required to reduce parasite viability by 50%, using Prism GraphPad software (version 8.2.1).

To assess the effectiveness of the crude extract, its fractions, isolated alkaloids, and Bz against intracellular amastigotes, *T. cruzi*-infected Vero cell cultures were treated for 72 h at 37 °C with specific concentrations: crude extract and fractions were treated with 0.22 to 500 μg mL^–1^, and isolated alkaloids and Bz at 0.041 to 100 μM. After treatment, cell monolayers were incubated with luciferin (300 μg mL^–1^), and luminescence was measured using a Glomax microplate reader. DMSO (<1%) was used as negative control. The IC_50_ and IC_90_ concentrations, corresponding to 50% and 90% inhibition of intracellular parasites, respectively, were determined using GraphPad Prism software. A minimum of three independent experimental assays were performed in duplicates.

#### *In Vitro* Combination Assays

The interactions between promising isolated alkaloids and their combinations with Bz, were assessed using an *in vitro* luminescence assay targeting amastigote forms of *T. cruzi* (Dm28c-Luc), which expresses luciferase. The assay was performed on previously infected Vero cell cultures, adhering to the described methodology. Maximum concentrations of each compound were established based on predetermined IC_50_ values, ensuring they were positioned around the midpoint within a series of seven dilutions. Combinations of the compounds followed fixed ratios of 5:0, 4:1, 3:2, 2:3, 1:4, and 0:5, with each mixture subjected to serial dilutions at 1:3 ratio prior to application on infected cell plates. After washing the cells with PBS to remove noninternalized parasites, they were treated with the compound mixtures for 72 h. Luminescence measurements quantified parasite viability upon the addition of *D*-luciferin (300 μg mL^–1^). IC_50_ values of each compound were calculated both for monotherapy and combination treatments, allowing the determination of the Fractional Inhibitory Concentration Index (FICI) according to the formula: FICI = IC_50_ (combination)/IC_50_ (isolated). The sum of the FICI (∑FICI) and the mean FICI (x∑FICI) were used to classify the type of pharmacological interaction: synergistic effects were defined as x∑FICI ≤ 0.5, additive interactions were characterized by 0.5 < x∑FICI < 4.0, and antagonistic effects were denoted by x∑FICI > 4.0. Additionally, isobolograms were constructed based on the FICI values to provide a graphical representation of the observed interactions.^22,23^

### *In Silico* Studies

#### ADME Predictions

The physicochemical properties of the isolated alkaloids were analyzed using DataWarrior software version 5.5.0.^24^ Chemical parameters, such as molecular weight (MW), lipophilicity (*c* Log *P*), solubility (*c* Log *S*), number of hydrogen bond donors (HBD) and acceptors (HBA), topological polar surface area (tPSA), and rotatable bonds (RB), were evaluated.

The compounds under study were designed in the Marvin sketch software v.19, ChemAxon, Budapest-Hungary (https://chemaxon.com/marvin, accessed on: August 14, 2025). The molecular geometry was initially optimized using molecular mechanics (MMFF94), followed by energy minimization by the semiempirical method Austin Model 1 (AM1)^25^ using Spartan 14 v.1 software, WaveFunction, Inc., Washington-USA (https://www.wavefun.com/, accessed on: August 15, 2025) and the most stable conformation of each compound was subjected to reverse docking simulations and subsequently molecular docking.

#### Preparation of the Compounds under Study

The chemical structures of the *C. scabrum* alkaloids under study (**C1**, **C2**, **C3**, and **C4**) were designed in two dimensions using MarvinSketch v.19.18 software (ChemAxon Ltd.: Budapest, Hungary, 2019) and saved in. sdf format. They were subsequently converted to their three-dimensional representation using Spartan’ 14 software (Wave function Inc.: Irvine, CA, 2014). Following this, a comprehensive conformational search was conducted to identify the lowest-energy conformation. The structure corresponding to the most stable conformer was then subjected to an energy minimization process using initially molecular mechanics methods, followed by a second minimization step using the semiempirical Austin Model 1 (AM1) method,^25^ ensuring the achievement of an energetically optimized geometry before its export in. sdf format for subsequent molecular docking steps.

#### Reverse Docking Prediction

Potential molecular targets were identified using reverse pharmacophore mapping using the PharmMapper platform (http://lilab.ecust.edu.cn/pharmmapper/, accessed on: August 15, 2025).^26–28^ The three-dimensional structure of the compound under study was submitted in. sdf format. The selected database was all targets, which contains pharmacophoric models derived from crystallographic structures of proteins belonging to various species. The analysis was configured to return the top 300 potential targets, ranked according to fit score and Z-score significance. The most relevant targets were selected and subsequently analyzed for their biological function, association with metabolic pathways, and possible correlations with the expected mechanism of action.

#### Molecular Docking Simulation

The 3D structure of the target Glyceraldehyde-3-phosphate dehydrogenase (GAPDH) of *T. cruzi* (PDB: 1ML3)^29^ as obtained from the Protein Data Bank (PDB) platform (https://www.rcsb.org/, accessed on: August 15, 2025). To validate the target predicted by reverse Docking methodology by the PharmMapper platform, due to the prediction result having had moderate validation, Docking was performed with a second target, this being the cysteine protease Cruzain of *T. cruzi* (PDB: 3I06),^30^ due to the scope and importance of this target for the survival of the parasite. The active sites of these proteins were identified using the template function between the target and the cocrystallized ligand. All water molecules were excluded from the enzyme structures, with mantio being the essential cofactors. Redocking was performed to validate the target and ligands under study, prior to performing the molecular docking simulation by evaluating the Root Mean Square Deviation (RMSD) metric, with a limit of up to 2 Å considered the acceptable value.^31^ The enzyme and compound structures were prepared using the default parameter settings of the software package (ligand evaluation: Internal ES, Internal H-bond, Sp2-Sp2 kinks, all checked; number of runs: 30 runs; algorithm: MolDock SE; maximum interactions: 1500; maximum population size: 50; maximum steps: 300; neighbor distance factor: 1.00; maximum number of returned poses: 5). The docking procedure was performed using GRID with a radius of 18 Å and a resolution of 0.30 to cover the ligand binding site of each protein. The grid box was applied to the center of the target site. The MolDock scoring algorithm was used, together with the MolDock search algorithm.^32^ Molegro Virtual Docker generated five poses for each molecule in the active site of each protein. The most stable pose, that is, the one with the lowest interaction energy, was selected and imported into Discovery Studio Biovia v.25, Vélizy-Villacoublay, France (https://www.3ds.com/products/biovia/discovery-studio, accessed on August 15, 2025) for visual inspection.

## Results and Discussion

### Isolated Compounds

Chromatographic purification of the leaves and bulbs of *C. scabrum* yielded four known alkaloids (Figure 1). Following separation and purification, one-dimensional ^1^H NMR spectra were recorded and compared with literature data (see Supporting Information) to confirm compound identities haemanthidine/6-*epi*-haemanthidine (**C1/2**), crinamine (**C3**) and reported for the first time for the spice hamayne (**C4**).

**Figure 1:**
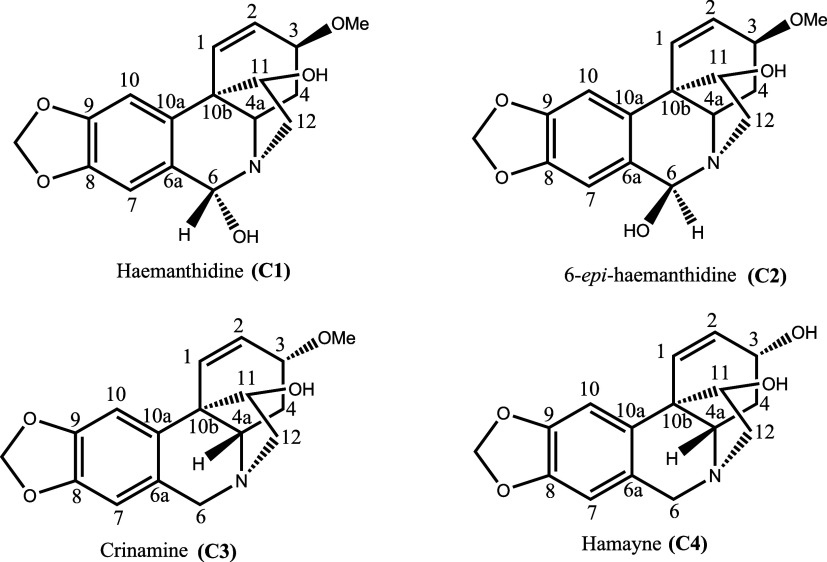
Alkaloids isolated from leaves and bulbs of *C. scabrum*.

### Biological Assays

#### Cytotoxicity and Anti-*T. cruzi* Activity

The exceptional antiamastigote activity of fraction III (Table 1), demonstrating potency comparable to Bz (IC_50_ = 0.88 ± 1.65 μg mL^–1^ vs 0.75 ± 0.07 μg mL^–1^) and unprecedented selectivity (SI > 568.2), aligns directly with its enrichment in bioactive Amaryllidaceae alkaloids. This chemotypic dominance explains its superior performance over other fractions, as prior studies validate the antiparasitic efficacy of structurally related alkaloids against *T. cruzi* amastigotes: haemanthamine (IC_50_ = 2.40 μM), lycorine (IC_50_ = 7.7 μM), and aulicine (IC_50_ = 55.5 μM).^18^ Critically, fraction III’s targeted action against intracellular amastigotes, responsible for chronic Chagas pathology. The observed potency strongly aligns with several proposed mechanisms for AAs, suggesting a mechanism involving mitochondrial disruption via the uncoupling of oxidative phosphorylation.^33^ Amastigotes are highly energy-dependent for their replication and survival within host cells.^17^ Therefore, if the alkaloids in the fraction act by altering mitochondrial membrane potential and uncoupling oxidative phosphorylation, leading to ATP depletion, as suggested for narcyclasine,^14^ this would devastate the amastigotes’ primary energy production pathway. Such a disruption in a metabolically active organism would be profoundly lethal.

**Table 1:** Cytotoxicity and Anti-*T. cruzi* Activity of *C. scabrum* Crude Extract and Polar Fractions^a^

	anti-*T. cruzi* activity mean ± SD (μg mL^–1^)						cytotoxicity (μg mL^–1^)
extracts/fractions	trypomastigotes			intracellular amastigotes			VERO
	IC_50_	IC_90_	SI	IC_50_	IC_90_	SI	CC_50_
E.MeOH	>500	>500	Nd	8.21 ± 1.65	48.4 ± 4.11	>60.9	>500
I	>500	>500	Nd	3.42 ± 0.40	9.27 ± 2.21	>146.2	>500
III	>500	>500	Nd	0.88 ± 0.08	3.71 ± 0.65	>568.2	>500
IV	>500	>500	Nd	85.29 ± 9.14	293.9 ± 17.1	>5.86	>500
Bz	4.76 ± 0.72 (18.3 μM)	>26.25 (100 μM)	>102	0.75 ± 0.07 (2.89 μM)	2.17 ± 0.31 (8.34 μM)	>666.7	>500 (1920 μM)

a Mean IC_50_ and IC_90_ values from three independent experiments ± standard deviation (SD); IC_50_: concentration that inhibits parasite viability by 50%; CC_50_: concentration that reduces Vero cell viability by 50%; Selectivity index (SI) = CC_50_ of Vero cells/IC_50_ of trypomastigotes and intracellular amastigotes of *T. cruzi*.

Furthermore, the AAs’ ability to inhibit key parasitic enzymes, such as trypanothione reductase or Cruzipain, could also be a significant contributor to the potent activity. Trypanothione reductase, for instance, is vital for maintaining the parasite’s redox balance,^34^ while Cruzipain is essential for its protein metabolism and host protein degradation.^35^ Inhibiting either of these enzymes would severely compromise the amastigote’s ability to proliferate and survive within its intracellular niche.^36,37^

While direct confirmation that AAs induce lethal oxidative stress by overwhelming the parasite’s antioxidant defenses remains to be explicitly demonstrated, the mitochondrial dysfunction triggered by some AAs could secondarily increase ROS production. Amastigotes, like other parasite forms, must maintain a delicate redox balance.^38^ An unmanageable increase in ROS, even if not the primary mechanism of action for AAs, could enhance their trypanocidal effect, exacerbating cellular and macromolecular damage. This concept of overwhelming the parasite’s antioxidant defenses is a known mechanism for Bz,^39^ suggesting a potential convergent pathway for the observed activity.

These processes selectively compromise amastigote survival without significant host cell cytotoxicity, positioning alkaloid-enriched fractions as promising scaffolds for overcoming the limitations of current monotherapies.

Conversely, none of the fractions and crude extract showed activity against trypomastigote forms, the circulating stage responsible for acute dissemination and vector transmission. This stage-specific selectivity parallels benznidazole’s own limitations, which primarily targets intracellular amastigotes.

To evaluate the specific contributions of the alkaloids haemanthidine/6-*epi*-haemanthidine (**C1/2**), crinamine (**C3**), and hamayne (**C4**) to the biological activity of fraction III, their bioactivity was assessed against *T. cruzi*. In the preliminary screening, these compounds were analyzed at a concentration of 20 μM to determine their overall activity against intracellular amastigotes (Figure 2). Thus, Vero cells were infected with the genetically modified *T. cruzi* Dm28c expressing luciferase (Dm28c-Luc) and treated for 72 h with **C1/2**, **C3**, and **C4** (20 μM). The results revealed that both **C1/2** and **C3** exhibited significant antiparasitic activity, achieving over 90% inhibition of intracellular parasite viability, comparable to that of Bz (Figure 2).

**Figure 2:**
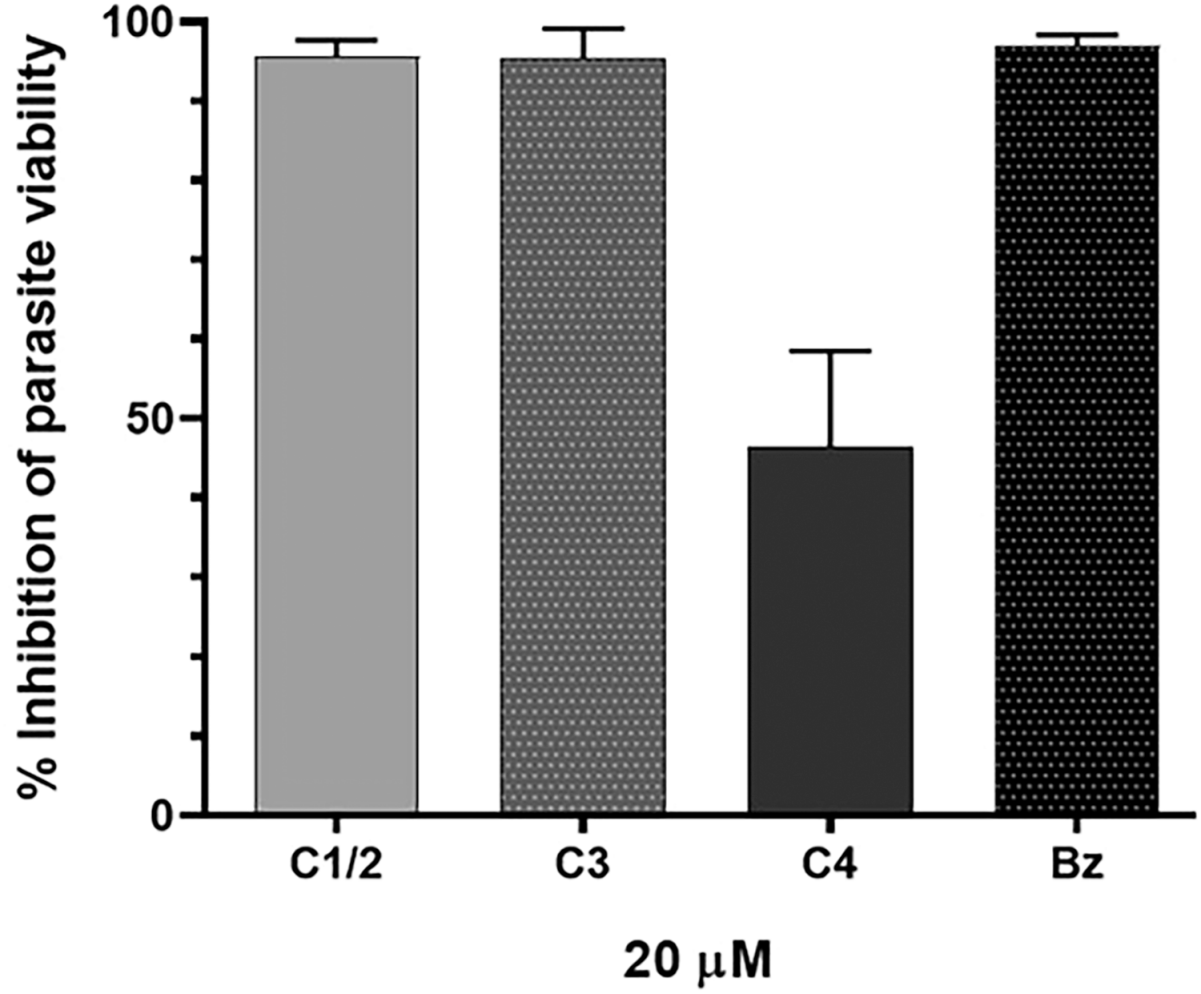
Effect of **C1/2**, **C3**, and **C4** on *T. cruzi* intracellular amastigotes. Vero cells infected by *T. cruzi* Dm28c-Luc (24 h) were treated for 72 h with 20 μM of isolated alkaloids, followed by luciferin addition and luminescent readout in a Glomax microplate reader. Data are presented as percentage inhibition of parasite viability.

Considering the role of both *T. cruzi* forms (trypomastigotes and amastigotes) in the ChD’s pathogenesis, the alkaloids’ activity on trypomastigotes was also evaluated (Table 2). The concentration–response curve of the alkaloids was determined between 0.41 to 100 μM to establish the IC_50_ values. All isolated alkaloids demonstrated activity against trypomastigotes. Particularly remarkable were compounds **C3** (IC_50_ = 9.28 ± 2.09 μM) and **C4** (IC_50_ = 5.63 ± 1.43 μM), which exhibited IC_50_ values approximately two to three times lower than the reference drug Bz (IC_50_ = 18.30 ± 2.78 μM). Importantly, the alkaloids exhibited no toxic effect on Vero cells, as indicated by their CC_50_ values exceeding 500 μM.

**Table 2:** Cytotoxicity and Anti-*T. cruzi* Effect of **C1**/**2**, **C3**, and **C4^a^**

	anti-*T. cruzi* activity mean ± DP (μM)						cytotoxicity
compounds	trypomastigotes			intracellular amastigotes			vero cells
	IC_50_	IC_90_	SI	IC_50_	IC_90_	SI	CC_50_
**C1/2**	26.65 ± 3.37	>100	>18.76	6.05 ± 0.53	12.40 ± 0.87	>82.64	>500
**C3**	9.28 ± 2.09	>100	>53.88	5.79 ± 0.17	13.21 ± 1.52	>86.35	>500
**C4**	5.63 ± 1.43	>100	>88.81	18.59 ± 1.39	41.05 ± 5.14	>26.90	>500
Bz	18.30 ± 2.78	>100	>27.32	2.89 ± 0.26	8.34 ± 1.19	>173	>500

a Mean IC_50_ and IC_90_ values from three independent experiments ± standard deviation (SD); IC_50_: concentration that inhibits parasite viability by 50%; CC_50_: concentration that reduces Vero cell viability by 50%; Selectivity index (SI) = CC_50_ of Vero cells/IC_50_ of trypomastigotes and intracellular amastigotes of *T. cruzi*.

The IC_50_ values of the purified alkaloids were also determined for intracellular parasites (Table 2). The **C1**/**2** and **C3** were the most effective compounds against intracellular amastigotes, with IC_50_ values of 6.05 ± 0.53 μM and 5.79 ± 0.17 μM, respectively. On the other hand, **C4** exhibited a low antiparasitic effect with an IC_50_ of 18.59 ± 1.39 μM. The selectivity index (SI) indicated that these compounds effectively target the intracellular parasites, with SI values exceeding 80 for the most active alkaloids (**C1**/**2** and **C3**).

Given that fraction III, which encompasses the purified alkaloids, exhibited the most significant antiparasitic activity, we proceeded to assess the effect of interaction between the most potent alkaloids, **C1**/**2** and **C3**, combined with one another and their individual combinations with Bz. The mean fractional inhibitory concentration (∑FICI) values obtained were 1.40 for the **C1**/**2**-Bz combination, 1.39 for **C3**-Bz, and 0.78 for **C1**/**2**-**C3** (Figure 3). These results indicate that all pairs of combinations tested exhibit an additive interaction, characterized by a combined effect equivalent to the sum of the individual effects of each compound. It is noteworthy that the combination of the **C1**/**2** and **C3** alkaloids was the combination that most closely resembled a synergistic profile (∑FICI ≤ 0.5).^40^ Extracts and alkaloids from the Amaryllidaceae family have already been reported for their potent synergistic effect on differents stages of *T. cruzi* when combined with Bz. For example, the alkaloids-rich extracts from *Hipperastrum* species proved to be a good source of active compounds. The main isolated alkaloid, montanine, exhibited potent dose-dependent activity against *T. cruzi* epimastigotes, showing an IC_50_ value of 0.55 μg mL^–1^, which is significantly lower than that Bz (IC_50_ = 4.58 μg mL^–1^).^41^ Crucially, the combination assay of montanine and Bz produced a potent synergistic effect (CI ≤ 0.169). This synergy allowed for a dose reduction of montanine and Bz by 9.4 and 15.8-fold, respectively. This favorable synergy led to 97% parasite inhibition at IC_50_ concentrations.^41^

**Figure 3:**
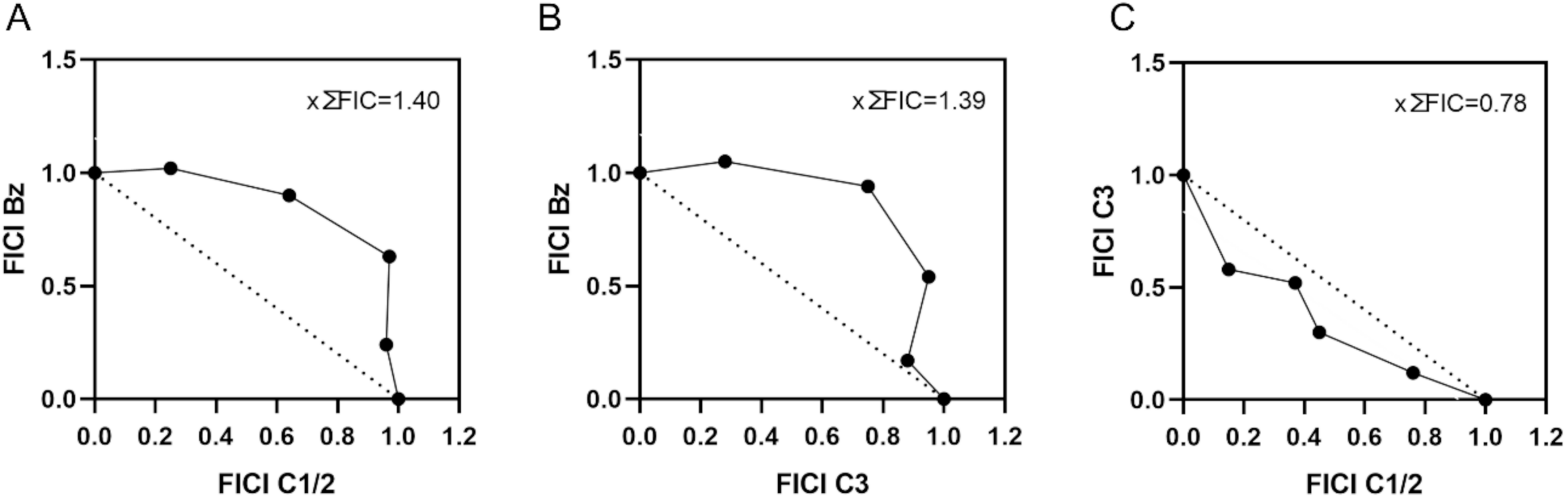
*In vitro* combined effect of the alkaloids and Bz against intracellular amastigotes. Isobologram plots for the **C1/2**–Bz (A), **C3**–Bz (B), and **C1/2**–**C3** (C) combinations were constructed based on the FICI values obtained for different ratios of the compounds. The mean ∑FICI values are represented in the graphs.

This synergistic pattern is also observed with candimine, another AA isolated from *Hipperastrum escoipense*. Similar to montanine, candimine demonstrated greater potency than Bz against amastigotes. However, its main potential lies in its high activity and selectivity against intracellular amastigotes, achieving a superior selectivity index (SI = 159.63) compared to that Bz (SI = 147.55). The combination of candimine and Bz revelated a potent synergism (CI = 0.08) specifically against amastigotes, which allows for a Bz dose reduction of up to 17.6-fold. Additionally, this same combination showed an antagonistic effect on Vero and HepG2 cells, a highly desirable finding, as it suggests a reduction host toxicity.^42^

Nevertheless, the additive interaction remains relevant, as it may enable a reduction in individual compound doses, thereby minimizing adverse effects and broadening the spectrum of activity in *in vivo* models of experimental *T. cruzi* infection.^43,44^ Theses findings reinforces the premise that AAs represent promising scaffolds for developing combined and less toxic treatments.

### *In Silico* Studies

#### AMDE Predictions

The use of computational tools in early phases of drug development is essential for predicting physicochemical properties that significantly impact pharmacokinetics and pharmacodynamics, particularly concerning oral bioavailability. Thus, we evaluated the physicochemical parameters of the isolated alkaloids (**C1/2**, **C3**, and **C4)** using DataWarrior software. The molecular weights of the analyzed molecules ranged from 287.31 to 317.34 g mol^–1^ (Table 3). Lipophilicity (*c* Log *P*), a key property involved in membrane permeability and protein binding, was also evaluated. The reported *c* Log *P* values for the alkaloids were between 0.53 and 0.74 (Table 3), indicative of low lipophilicity. Furthermore, solubility predictions suggest good aqueous solubility, with *c* Log *S* between −3.07 and −2.75, which is vital low solubility can adversely affect systemic drug concentrations.^45^ The potential for ligand-target interaction was examined through the assessment of hydrogen bond donors (HBD) and acceptors (HBA). All alkaloids registered HBA values of either 1 or 2. All alkaloids had an HBA value of 1 or 2. The topological polar surface area (tPSA), ideally not exceeding 140 Å, reached a maximum of 71.39 (Table 3), suggesting favorable permeability across biological barriers. Distinct profiles of rotatable bond (RB) were observed, peaking at a maximum of 2, suggesting good compound flexibility. All alkaloids exhibited promising drug-likeness predictions. Collectively, the data indicates that these compounds adhere to Lipinski’s rule of 5 (MM < 500, *c* Log *P* < 5, HBD < 5, and HBA < 10) and suggest a favorable profile for oral bioavailability.

**Table 3:** *In Silico* Physicochemical Properties of Isolated Alkaloids^a^

compounds	MW	*c* Log *P*	HBD	HBA	Log *S*	tPSA	RB	drug-likeness
**C1**/**C2**	317.34	0.74	2	6	–2.75	71.39	1	42.10
**C3**	301.34	0.96	1	5	–3.06	51.16	1	42.58
**C4**	287.31	0.53	2	5	–2.94	62.16	0	43.12

a Physicochemical properties: molecular weight (MW), lipophilicity (*c* Log *P*), number of hydrogen bond acceptors (HBA) and donors (HBD), water solubility (*c* Log *S*), topological polar surface area (tPSA), rotatable bonds (RB), and drug-likeness.

#### Reverse Docking Prediction

Molecular target prediction using the PharmMapper platform yielded a total of 300 potential targets. The analysis was prioritized based on fit score and Z-score, selecting targets with the highest relevance in the platform’s main scores and those related to the *T. cruzi* species. The results demonstrated that for the four compounds under study, a potential target related to ChD was identified: GAPDH of *T. cruzi* (PDB: 1ML3).

It is important to mention that the target GAPDH of *T. cruzi* is a central enzyme of glycolytic metabolism, being responsible for the conversion of glyceraldehyde-3-phosphate into 1,3-bisphosphoglycerate with generation of NADH, being essential for the energy production of the parasite.^46^ In addition to its role in energy metabolism, due to its presence in glycosomes, GAPDH can participate in additional functions, such as redox regulation and interactions with the cytoskeleton, reinforcing its relevance as a therapeutic target in the fight against ChD, as it contributes with glycolytic activity to sustain motility and survival outside the host cell.^38^ The scores corresponding to the reverse docking scores for the compounds under study against the enzyme GAPDH of *T. cruzi* (PDB: 1ML3) are shown in Table 4.

**Table 4:** Reverse Docking Score Prediction of Study Compounds for the GAPDH of *T. cruzi* (PDB: 1ML3) According to the PharmMapper Platform

compounds	fit-score	normalization score	Z-score
**C1**	3.469	0.693	2.757
**C2**	3.469	0.693	2.689
**C3**	3.213	0.642	1.807
**C4**	3.229	0.645	1.850

The data in Table 4 demonstrate that the Fit-score values for the four compounds under study were above 3, being considered a moderate and acceptable fit, while the Z-score values ranged from 1.807 to 2.757, indicating that the result obtained is not random and can be accepted. The definition of a moderate and plausible result is confirmed by the values referring to the Normalization score metric, since the values obtained were above 0.6. Compounds **C1** and **C2** presented the highest scores in the two metrics under study, corresponding to 4.469 (Fit-score), 0.693 (Normalization score), and 2.757 (Z-score) for compound **C1** and 3.469 (Fit-score), 0.693 (Normalization score), and 2.757 (Z-score) for compound **C2**. For compound **C3**, metrics of 3.213 (Fit-score), 0.642 (Normalization score), and 1.807 (Z-score) were observed. Compound **C4** presented scores of 3.229 (Fit score), 0.645 (Normalization score), and 1.850 (Z-score).

Figure 4 demonstrates the pharmacophoric model of the compounds under study with the target GAPDH of *T. cruzi* (PDB: 1ML3), demonstrating the presence of two hydrophobic regions corresponding to the benzene ring fused to the pyridine ring (blue circle), a negative region (red circle) and two hydrogen acceptors (pink circle), these corresponding to the 1,3-benzodioxole group, indicating that their interaction with the target occurs mainly by fitting into nonpolar pockets to exert their activity.

**Figure 4:**
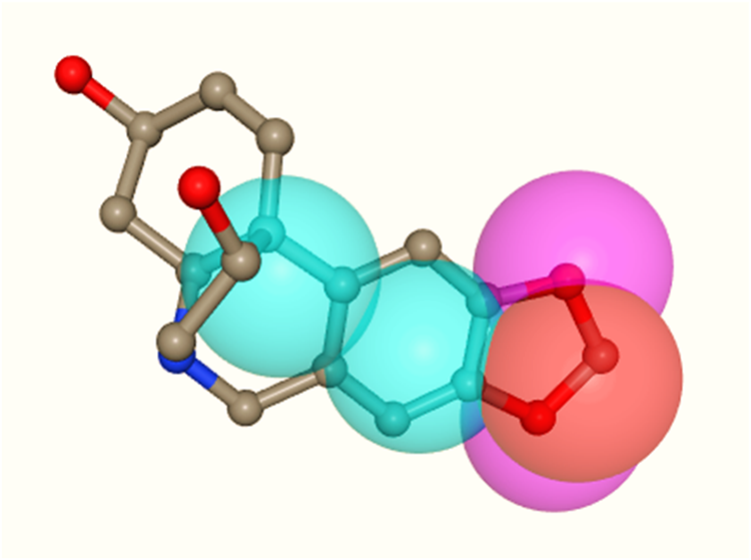
Pharmacophoric representation of the compounds under study with the target GAPDH of *T. cruzi* (PDB: 1ML3) according to the PharmMapper platform. Caption: Hydrophobic regions (blue circles), negative region (red circle) and hydrogen acceptors (pink circle).

#### Molecular Docking Simulation

Molecular docking was used to investigate potential targets associated with the antitrypanocidal mechanism of action, as well as to validate both the pharmacophoric model and the reverse docking screening performed by the PharmMapper platform. The affinity of the complexes formed by the study compounds with the target GAPDH of *T. cruzi* (PDB: 1ML3) was evaluated, based on the reverse docking proposal. However, as the pharmacophore prediction was considered moderate, a molecular docking simulation was also performed with the cysteine protease Cruzain of *T. cruzi* (3I06) to validate the model proposed in the reverse docking simulation.

It is important to mention that Cruzain of *T. cruzi* (PDB: 3I06) is an essential cysteine protease for *T. cruzi*, as it participates in obtaining nutrients, differentiation of evolutionary forms, evasion of the host’s immune response and maintenance of infectivity, being considered one of the main therapeutic targets for the development of new drugs against ChD.^47^

To evaluate the affinity of the compounds to the targets, the cocrystallized ligands present in the crystallographic structures (PDB ligands) of the respective enzymes were used as control parameters. These ligands correspond to a phosphonic acid derivative for the enzyme GAPDH of *T. cruzi* (PDB: 1ML3) and a carbonitrile derivative for Cruzain (PDB: 3I06), both experimentally validated. The structures used were selected considering the proximity between the molecular mass of the ligands and that of the compounds evaluated, since the MolDock Score algorithm incorporates the number of heavy atoms and the molecular weight in the energy calculation.^32^ Thus, the docking values were used exclusively to guide the interpretation of possible binding modes and to suggest potential targets compatible with the observed biological effects, and were not applied to establish quantitative comparisons of affinity between structurally distinct molecules, this approach is based on the inherent limitations of molecular docking methods, which include simplifications of energy potentials and restrictions on conformational exploration.^48^

The results of the molecular Docking simulations for the complexes formed between the compounds under study and the targets GAPDH of *T. cruzi* *(*PDB: 1ML3) and Cruzain of *T. cruzi* (PDB: 3I06), respectively, can be seen in Table 5, with the lowest binding energy value being considered as the result of greatest affinity according to the energy score of the MolDock Score algorithm.

**Table 5:** Binding Energy and Root Mean Square Deviation (RMSD) Values for the Complexes under Study According to the Energy Score of the MolDock Score Algorithm^a^

	GAPDH (PDB: 1ML3)		Cruzain (PDB: 3I06)	
compounds	binding energy	RMSD	binding energy	RMSD
**C1**	–51.731	0.34	–66.985	1.43
**C2**	**–66.619**		–67.830	
**C3**	–54.362		–73.966	
**C4**	–48.134		–73.14	
**PDB ligand**	–45.047		**–106.068**	

a Legend: The lowest binding energy value is highlighted in bold.

Prior to carrying out the molecular Docking simulations, redocking was performed in order to evaluate whether there are structural differences between the cocrystallized ligand and its most stable pose, this being evaluated by the value of the Root Mean Square Deviation metric, with 2Å being considered the widely accepted value for validating the method.^31^ The results available in Table 5 demonstrate that the RMSD values for the complexes under study followed the acceptability range, validating the aforementioned method.

The analysis of the binding energy values demonstrates that the pharmacophoric model proposed in the PharmMapper platform was validated, corroborating the result predicted through the reverse docking methodology, since for the target GAPDH of *T. cruzi* (PDB: 1ML3), the complexes formed presented lower binding energy values when compared to the PDB ligand (Phosphonic acid derivative), with the compound with the lowest binding energy value corresponding to the **C2** derivative (−66,619), followed later by the **C3** (−54,362), **C1** (−51,731) and **C4** (−48,134) complexes, while the PDB ligand (Phosphonic acid derivative) presented a binding energy value corresponding to −45,047. For the enzyme Cruzain of *T. cruzi* (PDB: 3I06), the compound with the lowest binding energy value corresponded to the PDB ligand (carbonitrile derivative), corresponding to −106.068, the complexes corresponding to the test compounds, respectively obtained the following binding energies: −73.966 (**C3**), −73.14 (**C4**), −67.830 (**C3**) and −66.985 (**C1**).

Table 6 demonstrates the molecular interactions observed in the complexes formed between the compounds under study and the target GAPDH of *T. cruzi* (PDB: 1ML3).

**Table 6:** Types of Interactions Formed between the Compounds under Study and Residues of the Target GAPDH of *T. cruzi* (PDB: 1ML3)­^a^

interactions	**C1**	**C2**	**C3**	**C4**	PDB ligand
alkyl	Cys 166	Cys166; His194			
carbon hydrogen bond		Asn335	Thr225	Asn335	
conventional hydrogen bond	**Thr226**; Arg249	**Thr226**; Arg249	Cys166; Arg249	Asn335; Cys166; **Thr226**; Arg249	Thr225; **Thr226**
covalent bond	**Cys166**		**Cys166**	**Cys166**	
pi-alkyl			Cys166; His194	Cys166; His194	
pi–pi stacked		His194		His194	
unfavorable acceptor–acceptor	His194				Ser247; Arg249

a Legend: Similar residues between the test compounds and PDB ligand (Phosphonic Acid derivative) are highlighted in bold.

Table 6 shows that the complexes of test compounds **C1**, **C2**, and **C4** with the target GAPDH of *T. cruzi* (PDB: 1ML3) presented interactions similar to the complex related to the PDB ligand, corresponding to residue Threonine 226, which represents an important catalytic residue for the target under study. A second important interaction is visualized in the complexes related to compounds **C1**, **C3**, and **C4** through the covalent interaction through residue Cysteine 166, which is a component of the enzyme’s active site and crucial for its inhibition.^29^

The molecular interaction maps of the compounds under study with the target GAPDH of *T. cruzi* (PDB: 1ML3) are shown in Figure 5, showing a correlation with the pharmacophoric model proposed by the PharmMapper platform, in which it is possible to observe the involvement of the benzene ring groups fused to the pyridine ring and the 1,3-benzodioxole group with the enzyme under study. Despite the hydrophobic contribution in the complexes, hydrogen bond-type interactions (dark green dashed line) established with the hydroxyl (OH) groups and with the nitrogen (N) and oxygen (O) atoms of the compounds are also observed, with lengths above 2.50 Å, indicating adequate proximity between donor and acceptor, as well as biological relevance. This phenomenon is observed through the Threonine 226 residue in compound **C1** with length of 2.94 and for the Arginine 249 residue in compounds **C2** (2.63), **C3** (2.50) and 2.58 (**C4**), demonstrating the effectiveness of the interaction.

**Figure 5:**
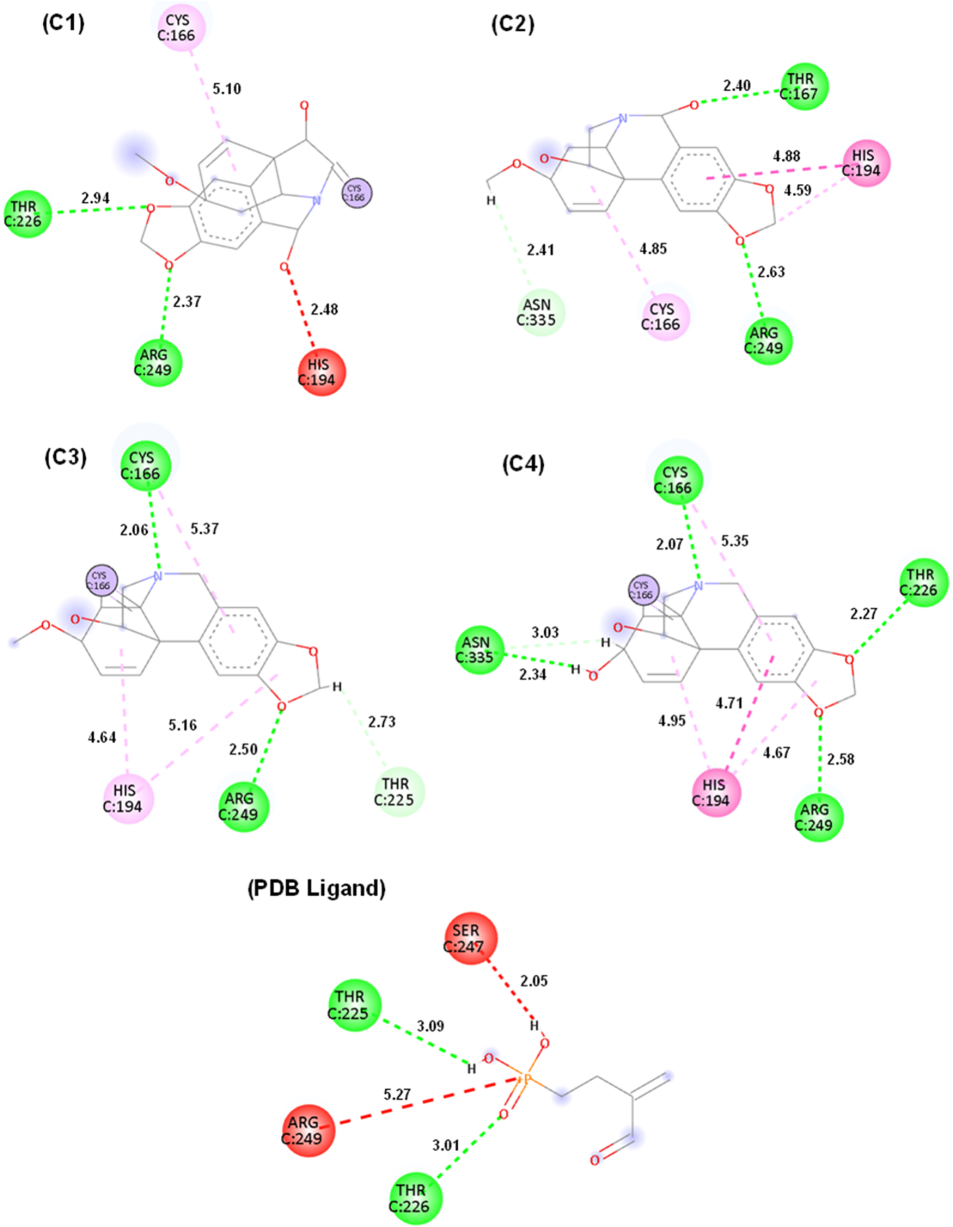
Molecular interaction maps of the complexes formed by compounds **C1**, **C2**, **C3**, **C4**, PDB ligand (Phosphonic Acid Derivative) with the target GAPDH of *T. cruzi* (PDB: 1ML3). Interactions: Conventional hydrogen interaction (dark green line), Carbon–hydrogen interaction (light green line), Covalent interaction (purple circle), Alkyl and pi-alkyl interaction (light pink line), Pi-Pi stacked interaction (dark pink line) and Unfavorable Acceptor-Acceptor (red line). Residues: Cysteine (Cys), Threonine (Thr), Arginine (Arg), Histidine (His), Asparagine (Asn) and Serine (Ser).

## Conclusions

This study establishes *C. scabrum* as a compelling source of antitrypanosomal agents, with its alkaloid-enriched Fraction III emerging as a particularly potent, low-toxicity alternative for chronic ChD. Fraction III demonstrated exceptional activity against intracellular *T. cruzi* amastigotes, rivaling benznidazole (Bz) while exhibiting unprecedented selectivity, a finding attributed to its high concentration of crinane-type alkaloids (haemanthidine/6-*epi*-haemanthidine [**C1**/**2**] and crinamine [**C3**]). Notably, Fraction III significantly outperformed purified alkaloids, underscoring enhanced efficacy driven by additive interactions within the phytochemical matrix. Critically, combination studies of the major alkaloids (**C1**/**2** and **C3**) with each other and with Bz revealed additive interactions. While the **C1**/**2**-**C3** combination showed the strongest effect, all interactions remained additive. This additive effect, combined with low cytotoxicity, positions *C. scabrum* as a strategic candidate for overcoming limitations of current monotherapies, as it may enable dose reduction of individual compounds, minimizing adverse effects and potentially broadening activity.

Through the integration of computational and *in vitro* approaches was possible to identify the potential bioactivity of the isolated alkaloids against *T. cruzi*. Two possible mechanisms of action of the alkaloids were identified, the inhibition of enzyme GAPDH and cysteine protease Cruzain. Moving forward, *in vivo* validation of Fraction III and its key alkaloid combinations in murine models is essential to confirm parasitological clearance and safety.

## Supplementary Material



## Notes

General remarks and comparative ^1^H NMR data with literature for compounds **C1**/**2**, **C3** and **C4** (PDF)
