# Supplementary material for: Haemanthidine-Containing Alkaloid Fraction from Crinum scabrum as a Natural Therapeutics for Chagas Disease
Source: ACS Omega. 2025 Dec 25;11(1):1908–18. doi: 10.1021/acsomega.5c10218 (PMC12809786; doi:10.1021/acsomega.5c10218)
Supplement: Supplementary file 1 [file ao5c10218_si_001.pdf]

Haemanthidine-Containing Alkaloid Fraction from *Crinum scabrum* as a Natural Therapeutics for Chagas Disease

Jennifer Blandón Pardo[1], Lorraine Martins Rocha Orlando[2], Leonardo da Silva Lara[2], Mirian Claudia de Souza Pereira[2], Natália Ferreira de Sousa[3], Luciana Scotti[3], Marcus Tullius Scotti[3].
Warley de Souza Borges[1]*
[1]Natural Products Research Group, Department of Chemistry, Federal University of Espírito Santo, UFES, Vitória-ES, Brazil.
[2]Cellular Ultrastructure Laboratory, Oswaldo Cruz Institute, Fiocruz, Rio de Janeiro - RJ, Brazil.
[3]Postgraduate Program in Bioactive Natural and Synthetic Products, Federal University of Paraíba, Campus I, Cidade Universitária, João Pessoa - PB, Brazil.
*warley.borges@ufes.br

General Remarks

Solvents of analytical grades with purity higher than 99.5% were purchase from Synth (Sao Paulo, SP, Brazil). The dehydration oven (Ethik Technology, São Paulo, SP, Brazil) was used. Rotavapor model R-3 (BUCHI, Valinhos, SP, Brazil) connected to a V-100 vacuum pump (BUCHI, Valinhos, SP, Brazil) was used. For open-layer chromatography, 5 × 30 cm and 5 × 28 cm glass columns packed with silica gel stationary phase (particle size of 0.04-0.063 mm and 25-40 μm) in hexane were used. NMR spectra were recovered on a Varian 400 MHz instrument (Palo Alto, USA) using deuterated chloroform ($CDCl_3$) as solvent and tetramethylsilane (TMS) as the internal standard both from Sigma-Aldrich (St. Louis, MO, USA). The chemical shift (δ) is in ppm and *J* values in hertz (Hz).

Table 1S. ^{1}H -NMR (400 MHz, $CDCl_3$) spectroscopic data for compound **1**/**2**, **3** and **4**, compared with literature values.

			δ **^{1}H , (*J* in Hz)**			
			**Compound**			
**No.**	**1 / 2**	Haemanthidine/6-*epi*- haemanthidine (400 MHz, $CDCl_3$)[1]	**3**	Crinamine (300 MHz, $CDCl_3$)[2]	**4**	Hamayne (200 MHz, $CDCl_3$)[3]
**1**	6.18-6.24 *m*	6.28-6.38 *m*	6.24 *bs*	6.22 *bs*	6.21 *bs*	6.19 *bs*
**2**	6.18-6.24 *m*	6.28-6.38 *m*	6.25 *bs*	6.22 *bs*	6.21 *bs*	6.19 *bs*
**3**	3.89-3.91 *m*	3.87 *m*	4.01-3.98 *m*	3.98 *m*	4.41 *d* (17.1)	4.35 *m*
**4**	-	-	2.04-2.16*m*	-	-	2.10 *m*
**4α**	2.17-2.24 *m* / 2.26-2.33 *m*	2.38 *ddd* (13.6, 13.6, 4.4) / 2.27 *br dd* (12.4, 4.8)	-	2.08 *m*	2.12 *dd* (11.2, 5.5)	-
**4β**	2.09-2.12 *m*/ 2.17-2.24 *m*	2.20 *dd* (13.6, 4.4) / 2.07 *dd* (13.6, 4.8)	-	2.08 *m*	2.05 *dd* (12.6, 2.4)	-
**4a**	3.79 *dd* (12.0, 6.0) / 3.33-3.37 *m*	3.73 *dd* (13.6, 4.8) / 3.28-3.36 *m*	3.27 *dd* (4.7, 13.3)	3.22 *dd* (13.3, 4.7)	3.40 *m*	3.25 *dd* (13.5, 4.5)
**6**	-	-	-	-	-	-
**6α**	- / 5.02 *s*	- / 5.16 *s*	3.69 *d* (16.8)	3.66 *d* (16.9)	3.69 *d* (16.2)	3.65 *d* (16.0)
**6β**	5.60 *s*/ -	5.87 *s* /-	4.31 *d* (16.8)	4.28 *d* (16.9)	3.98 ddd (6.7, 3.5, 1.2)	4.30 *d* (16.0)
**7**	6.96 *s*/ 6.82 *s*	6.95 *s* / 6.80 *s*	6.49 *s*	6.47 *s*	6.47 *s*	6.47 *s*
**10**	6.73 *s*/ 6.75 *s*	6.76 *s* / 6.78 *s*	6.80 *s*	6.79 *s*	6.80 *s*	6.81 *s*
**11**	-	-	3.96-4.02 *m*	-	4.41 *dd* (10.0, 6.2)	4.00 *m*
**11 endo**	3.91-3.93 *m*	3.96 *m*	3.98-4.01 *m*	3.92 *m*	-	-
**12 endo**	3.33-3,37 *m*/ 4.21 *dd* (14.4, 6.8)	4.26 *dd* (14.0, 6.8) / 3.28 - 3.36 *m*	3.31 – 3.42 *m*	3.40 *m*	3.33 *dd* (13.9, 3.6)	3.35 *m*
**12 exo**	3.03 *dd* (17.6, 6.4) / 3.33-3.37 *m*	3.09 *dd* (14.4, 2.4) / 3.28 - 3.36 *m*	-	3.40 *m*	-	-
**OCH_2O**	5.89 *d* (1.4) / 5.91 *d* (1.4)	5.91 2d (1.6) / 5.93 2d (1.3)	5.90 *s*	5.86 *s*	5.90 *dd* (1.6,1.2)	5.90 *s*
**OCH_3**	3.39 *s*	3.36 *s* / 3.34 *s*	3.40 *s*	3.40 *s*	-	-

REFERENCES

(1) Bessa, C. D. P. B.; De Andrade, J. P.; De Oliveira, R. S.; Domingos, E.; Santos, H.; Romão, W.; Bastida, J.; Borges, W. S. Identification of Alkaloids from *Hippeastrum aulicum* (Ker Gawl.) Herb. (Amaryllidaceae) Using CGC-MS and Ambient Ionization Mass Spectrometry (PS-MS and LS-MS). *J. Braz. Chem. Soc.* **2017**, *28* (5). https://doi.org/10.21577/0103-5053.20160234.

(2) Likhitwitayawuid, K.; Angerhofer, C. K.; Chai, H.; Pezzuto, J. M.; Cordell, G. A. Cytotoxic and Antimalarial Alkaloids from the Bulbs of *Crinum amabile*. *J. Nat. Prod.* **1993**, *56* (8), 1331–1338. https:// doi: 10.1021/np50098a017.

(3) Viladomat, F.; Bastida, J.; Codina, C.; Campbell, W. E.; Mathee, S. Alkaloids from *Brunsvigia Josephinæ*. *Phytochemistry* **1994**, *35* (3). https://doi.org/10.1016/S0031-9422(00)90611-3.
